# Coffee Disease Visualization and Classification

**DOI:** 10.3390/plants10061257

**Published:** 2021-06-21

**Authors:** Milkisa Yebasse, Birhanu Shimelis, Henok Warku, Jaepil Ko, Kyung Joo Cheoi

**Affiliations:** 1 Department of Computer Engineering, Kumoh National Institute of Technology, Gumi 39177, Korea; milkisa@kumoh.ac.kr (M.Y.); nonezero@kumoh.ac.kr (J.K.); 2 Artificial Intelligence Center (AIC), Addis Ababa 2Q92+88, Ethiopia; breeshime@gmail.com; 3 Department of IT-Bio Convergence System, Electronics Engineering, Graduate School, Chosun University, Gwangju 61452, Korea; heni1032.tegegn@gmail.com; 4 Department of Computer Science, Chungbuk National University, Cheongju 28644, Korea

**Keywords:** coffee disease classification, coffee disease visualization, deep learning, Grad-CAM, Score-CAM

## Abstract

Deep learning architectures are widely used in state-of-the-art image classification tasks. Deep learning has enhanced the ability to automatically detect and classify plant diseases. However, in practice, disease classification problems are treated as black-box methods. Thus, it is difficult to trust the model that it truly identifies the region of the disease in the image; it may simply use unrelated surroundings for classification. Visualization techniques can help determine important areas for the model by highlighting the region responsible for the classification. In this study, we present a methodology for visualizing coffee diseases using different visualization approaches. Our goal is to visualize aspects of a coffee disease to obtain insight into what the model “sees” as it learns to classify healthy and non-healthy images. In addition, visualization helped us identify misclassifications and led us to propose a guided approach for coffee disease classification. The guided approach achieved a classification accuracy of 98% compared to the 77% of naïve approach on the Robusta coffee leaf image dataset. The visualization methods considered in this study were Grad-CAM, Grad-CAM++, and Score-CAM. We also provided a visual comparison of the visualization methods.

## 1. Introduction

Ethiopia is the birthplace of coffee and the current source of the best coffee in the world [1]. Today, coffee is grown in more than 50 countries worldwide and is consumed on all continents. For Ethiopia, coffee is the largest export good, accounting for 20–25% of the total foreign exchange earnings. At least 15 million people rely on coffee for their livelihood [2]. While Ethiopia has a rich coffee heritage, its market share has not yet been fully developed [3]. More than 80 percent of the coffee growers are peasant farmers. Entire communities depend on coffee for their survival, with small farmers fighting to stay alive while facing growing environmental challenges [4]. The average coffee yield in the country is generally low. This is partly due to diseases and the limited use of advanced technologies. The method used for the supervision of diseases is observation through the naked eye, which is time-consuming and expensive and requires considerable expertise. Therefore, it is important to automatically identify the diseases without the need for experts. Object detection can act as a powerful tool in disease detection [5].

In the past few years, deep learning, object detection, and image classification have made tremendous advances. Krizhevsky proposed a deep convolutional neural network that achieved high accuracy in the classification of images into 1000 possible categories [6]; since then, it has revolutionized most aspects of computer vision. The VGG network from the visual geometry group is the most notable one. It achieved a remarkable result of 93.2% top-5 accuracy and 76.3% top-1 accuracy on the ImageNet test set [7].

However, it is sometimes difficult to obtain accurate ground truth labels for many tasks because of the cost of data labeling. Thus, in 2015, Maxime Oquab proposed a weakly supervised learning strategy using a convolutional neural network to reduce the annotation burden [8]. A weakly supervised learning approach uses partially labeled or annotated data when training a machine learning model. Thus, weak supervision is more applicable and desirable for deep learning techniques [9]. This observation has sparked many weakly supervised learning studies [10,11,12,13], while other studies have attempted semi-supervised anomaly detection using autoencoders [14].

Deep neural networks have made great achievements; however, a deeper understanding of the computations they perform at their intermediate layers remains limited. Some researchers have developed techniques to visualize and localize saliency maps: Zeiler and Fergus proposed a novel visualization technique that provides insight into the top operation of convolutional neural network (CNN) [15]; Zhou proposed learning deep features for discriminative localization [16]; Yoo designed multi-scale pyramid pooling for deep convolutional representation [17]; Jo and Yu improved localization by matching partial and full features [18].

Recent advances in computer vision and machine learning have made it possible to automate disease diagnosis. A wide range of disease detection systems that use convolutional neural networks have been published recently, Kumer presented the use of a pre-trained model for the detection and classification of healthy and defected coffee plants [19]. Giuliano L. Mansoa presented a method that identifies leaves from images by using a segmentation algorithm, separating them from the background, and finally applying an artificial neural network trained using an extreme learning machine [20]. In contrast, Shanwen Zhang developed a novel approach to identifying cucumber diseases by segmenting diseased leaf images by K-means clustering, examining the shape and color of the diseased leaf lesions, and classifying diseased leaf images using sparse representation [21]. Enhanced visual attention Guided Deep Neural Networks for Image Classification has been proposed by Yeh by learning feature maps that highlight salient regions and weaken meaningless connected layers [22].

Many state-of-the-art methods already exist for plant disease classification and detection [23,24,25,26,27] and defect detection in general [28,29,30]. However, to the best of our knowledge, there are only a few studies on coffee disease detection and they focus only on classifying healthy and non-healthy leaves using transfer learning [31] or using an annotated bounding box for detection [32]. Nevertheless, these deep models are seen as “black box” approaches, and researchers face a lot of trial and error when developing a satisfactory CNN model. Even accuracy can be inflated if the model overfits the data. Visualization can help open the black box and see what is happening in the model. Therefore, in this paper, we propose a method for visualizing coffee disease classification using well-established visualization techniques. The visualization techniques considered in this study were Grad-CAM, Grad-CAM++, and Score-CAM.

To summarize, our contributions are as follows:

1. We present a guided approach that achieved 98% accuracy in coffee disease classification. Further, in this study, we provide visualization of coffee disease, which exclusively highlights the region responsible for classification.
2. In this study, we implement three visualization approaches: Grad-CAM, Grad-CAM++, and Score-CAM. We also provided a visual comparison of those approaches.
3. In this study, we demonstrate the relevance of visualization in coffee disease classification. In support of our argument, we present two models and compare their accuracy and visualization results. By comparing the naïve approach and guided approach, this paper will provide new researchers with insights into the factors to consider when applying visualization and classification.

The remainder of this article is organized as follows. In Section 2, we briefly review the visualization methods and present two network architectures to demonstrate the importance of visualizing coffee disease classification. In Section 3, we present the experimental results and a visual comparative analysis of the visualization methods. The conclusion follows in Section 4.

## 2. Materials and Methods

### 2.1. Visualization Method

In this section, we briefly review the well-established visualization techniques. This paper implemented three visualization methods, Grad-CAM, Grad-CAM++, and Score-CAM.

#### 2.1.1. Grad-CAM

Grad-CAM is a technique for visualizing important regions for available classes using guided propagation. It uses the gradient of any targeted class, passing into the final CNN layer to highlight important regions in the image for prediction [33]. Grad-CAM computes the gradient with respect to the feature map of a convolutional layer. To compute Grad-CAM, the gradient of the score for class c is computed first, with respect to a feature map activation *A^k^* of a convolutional layer, that is *∂y^c^/∂A^K^*. These gradients are global-average-pooled over the width and height dimensions (indexed by *i* and *j*, respectively) to obtain the neuron importance weights *w* (Equations (1) and (2)):

(1) $$w_{k}^{c}=\frac{1}{z}\;\sum_{i}\sum_{j}\frac{\partial y^{C}}{\partial A_{ij}^{K}}$$

In addition, the heatmap of Grad-CAM is computed using a combination of feature maps, followed by relu:

(2) $$L_{\mathrm{Grad}-\mathrm{CAM}}=\mathrm{relu}\left(\underset{k}{\sum }w_{k}^{c}A^{K}\right)$$

**Figure 1:**
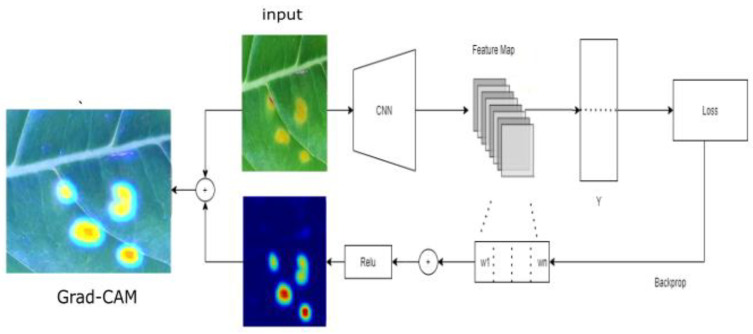
Block diagram of Grad-Cam on coffee disease detection.

Grad-CAM calculates the effect of each area of the image on the final output based on the gradient of the parameter of the final convolutional layer and calculates the degree of influence, represented by a heatmap. We used Grad-CAM to exclusively highlight the defective region. Additionally, we provided an emphasized Grad-CAM by calculating the sigmoid heatmap before adding the input image. Figure 1 shows the architecture of Grad-CAM, where y is the score of the class, and w is the neuron importance.

#### 2.1.2. Grad-CAM++

**Figure 2:**
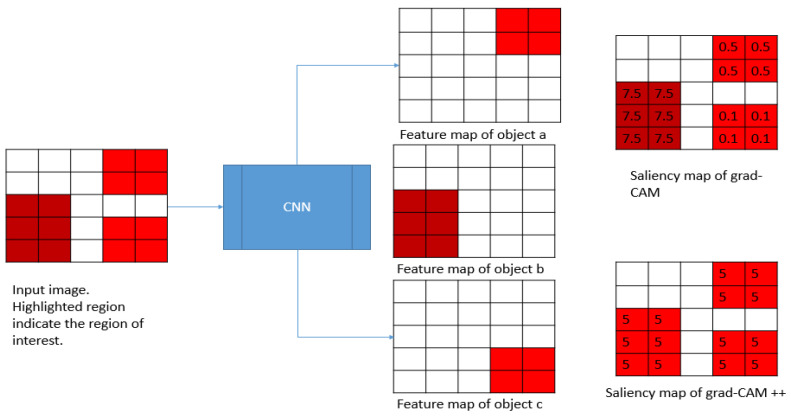
The intuition behind Grad-CAM.

Grad-CAM++ is derived from Grad-CAM. Grad-CAM++ provides improved visual explanations of CNN model predictions, thereby providing better localization of objects and explanations of the occurrences of multiple objects in a single image [34]. In Grad-CAM, if there are multiple objects with slightly different views or orientations inside the box, the feature map weights are different. In contrast, Grad-CAM++ highlights all relevant input regions equally. Figure 2 shows the weight combination of the Grad-CAM and Grad-CAM++. The values in each pixel on the saliency map represent the levels of intensity at that point.

It may be that the feature map with lesser weight becomes insignificant and fades away in the final saliency map; to solve this issue, Grad-CAM++ takes the weighted average of the pixel-wise gradients; $\alpha_{ij}^{kc}$ is the weighting coefficient for the pixel-wise gradient Equation (3).

(3) $$w_{k}^{c}=\underset{i}{\sum }\underset{j}{\sum }\alpha_{ij}^{kc}.relu\left(\frac{\partial y^{C}}{\partial A_{ij}^{K}}\right)$$

#### 2.1.3. Score-CAM

In gradient-based methods, the gradient of a target class is back-propagated to the input layer to highlight the region of the sample that is highly relevant to the prediction. In contrast, with Score-CAM, instead of dependence on gradients for the weight of each activation map, each activation map is obtained by passing its forward-passing score on the target class [35]. The working process of Score-CAM involves two phases. Step 1 is the extraction of activation maps, where each activation serves as a mask on the original image and calculates its forward-passing score to the target class. Step 2 is the linear combination of score-based weights and activation maps.

### 2.2. Visualization of Coffee Disease

In this section, we present a visualization of coffee disease to illustrate how its classification distinguishes between healthy and unhealthy leaves. We built deep learning models to classify and localize the defective regions. Visualization can also be a useful tool to help find issues in the learning process and even provide guidance on how to fix them. Grad-CAM, Grad-CAM++, and Score-CAM were used as visualization tools in this study. To illustrate our point, we developed two models for classifying coffee diseases: the naïve approach and the guided approach.

#### 2.2.1. Coffee Leaf Images Dataset

**Figure 3:**
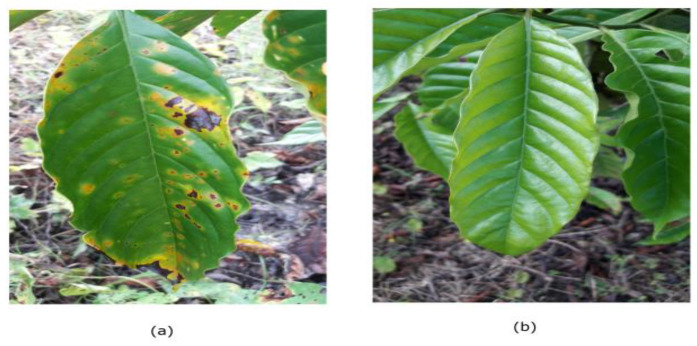
Robusta coffee leaf images dataset. (**a**) non-healthy image; (**b**) healthy image.

Our experiment was conducted using the Robusta coffee leaf image dataset (RoCoLe) [36]. The dataset consists of 1560 Robusta coffee leaf images with visible spots for non-healthy cases and healthy images. Images that are not healthy were infected by coffee rust. Coffee leaf rust, discovered at the end of the 19th century in Brazil, is one of the most destructive diseases of coffee plants in the world. Despite effective and integrated approaches using fungicides and resistant varieties developed to control rust, coffee leaf rust continues to have a debilitating effect on coffee growth [37]. Some of the symptoms of coffee rust include yellow spots on top of the leaf. Sometimes the spots may expand into larger round spots that turn bright orange to brown with a yellow border. Figure 3 shows a sample image of the coffee leaf dataset.

#### 2.2.2. Naïve Approach

**Figure 4:**
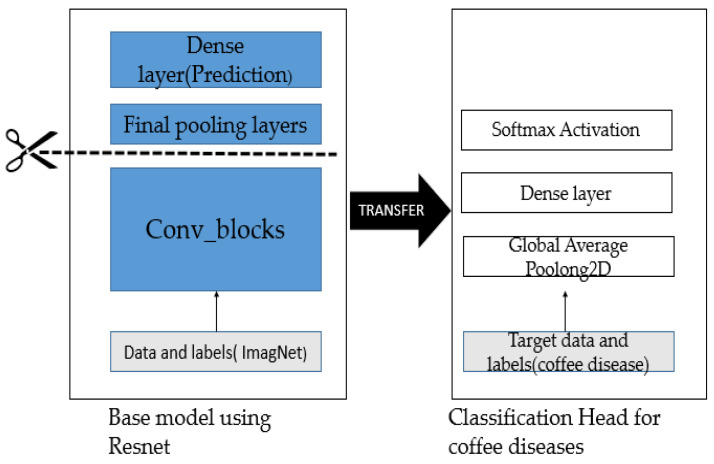
Deep Neural Network model (DNN-model).

**Figure 5:**
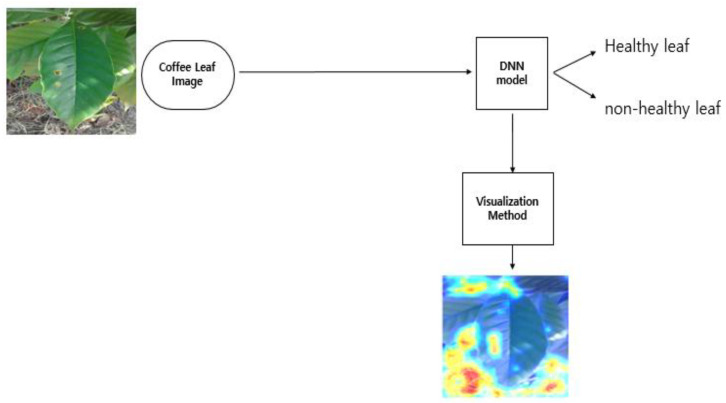
The pipeline of the naïve approach.

In the naïve approach, the network was trained using only original images from the dataset. We used ResNet as the backbone of our model [38]. The final pooling and prediction layers of Resnet are removed, while Global Average Pooling and dense output layers are added (Figure 4). The dense layers served as the classification layer. We used categorical cross-entropy as a loss function for our model. It compares the distribution of our predictions with the ground truth. The probability of the true class is kept as 1, and that of the false class is 0. We set the training hyperparameters as follows: the batch size was 32 and the RMSprop optimizer with an initial learning rate of 0.0001 was used. Grad-CAM was selected as the visualization tool. Figure 5 illustrates the naïve approach.

#### 2.2.3. Guided Approach

**Figure 6:**
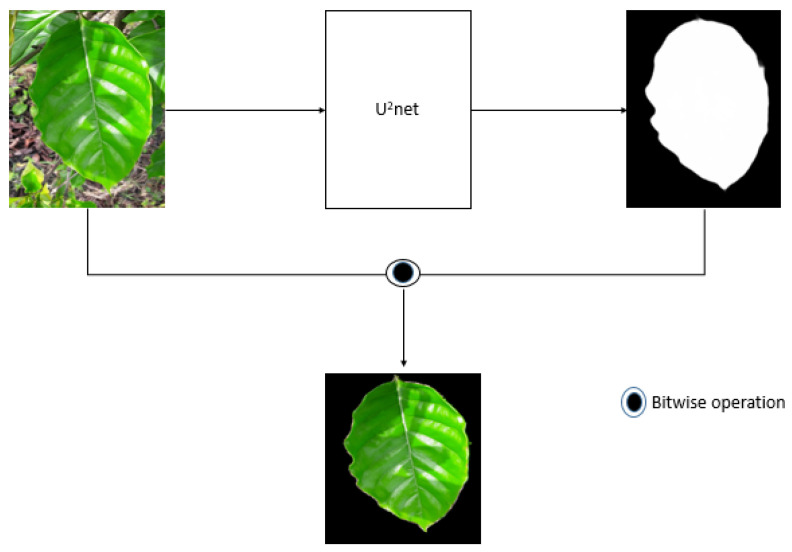
Segmented images using u^2^net.

A neural network has difficulty focusing only on the region of interest without any guidance. The naïve approach acquires too much functionality by learning commonalities that are not related to the disease, which can lead to overfitting. For example, by focusing on background soil structure. Therefore, we changed the background into something more meaningful and easier to analyze. We segmented the region of interest (leaf) from noise backgrounds like soil and small leaves, to guide the neural network. We used u^2^net to segment the images. The u^2^net is a powerful unsupervised neural network for salient object detection. The architecture of our u^2^net was a two-level nested U-structure. We used the pretrained u^2^net provided by the authors [39]. A pretrained u^2^net generates a corresponding masked image from the original input. Taking the masked image, we performed a bitwise operation between the original and segmented images (as shown in Figure 6).

**Figure 7:**
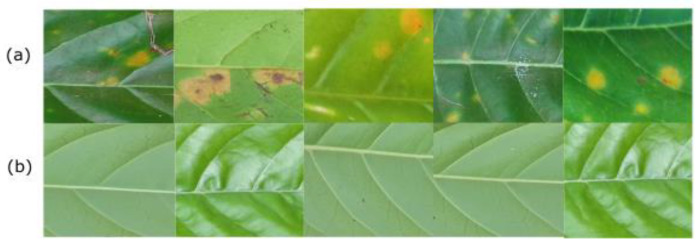
This figure shows a sample image of extracted patches. (**a**) non-healthy patch image; (**b**) healthy patch.

We also noticed that a rust spot could be quite small. For the model to work properly, attention must be paid to the smallest details when working with small local features. Such type of small details can be dealt with by extracting small patches from the segmented images. Our base dataset was obtained by extracting 4000 image patches from the segmented dataset. The extracted small patches have dimensions of 128 × 128 pixels. Approximately 80% of our dataset were used to train the models, while the remaining 20% were used to test them. Figure 7 shows the extracted patches.

**Figure 8:**
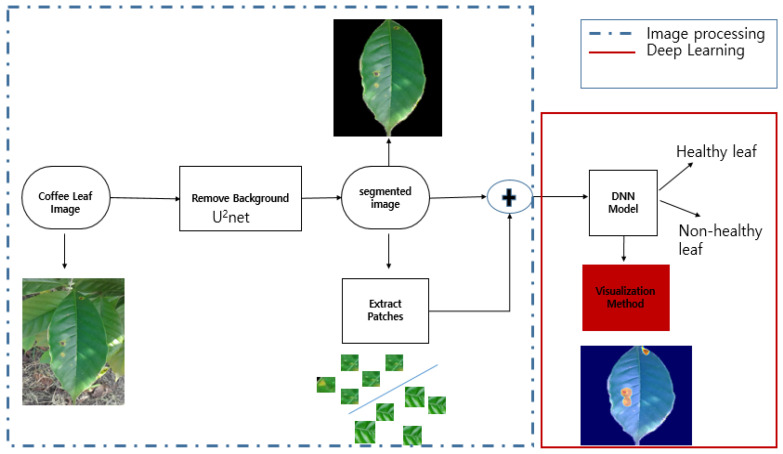
The pipeline of the proposed approach.

By segmenting the image, we can isolate only the region of interest, which will help guide the network. Additionally, by extracting patches, we can narrow the focus of the network to the rusty areas and increase our dataset as well. As a result, the proposed method (as shown in Figure 8) uses both segmented images and extracted patches as inputs for training. All images are resized to 224 × 224 and then, fed into the DNN model. We kept the DNN model similar to the naïve approach.

## 3. Results and Discussion

### 3.1. Naïve Approach

**Figure 9:**
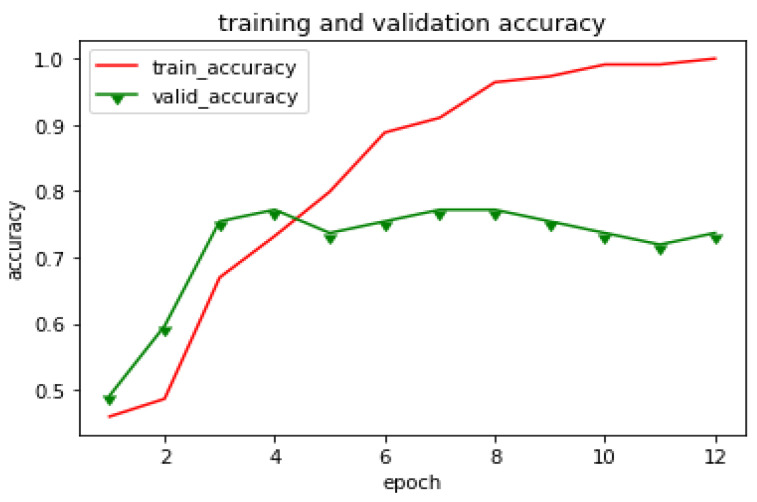
Training and test accuracy of naïve approach.

In this experiment, the network was trained using only the original images. Approximately 80% of the raw dataset was used for training, and the other 20% were used for testing. Figure 9 shows that the model achieved 99% accuracy in the training dataset. However, it performs poorly in terms of test accuracy. This model fails to generalize well from our training data to unseen data. This is called overfitting. Overfitting usually occurs when a model fits too closely to a limited dataset.

**Figure 10:**
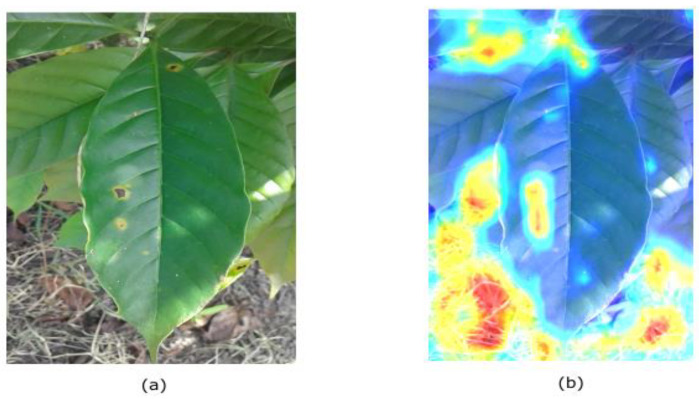
Visualization Result on naïve approach (**a**) input image; (**b**) highlighted image.

Visualizing our model can help resolve this problem. Visualization can serve as a guide for determining ways to improve the classifier. For example, Figure 10 depicts the visualization results of non-healthy leaves. Regions that are the most responsible for classifying objects are highlighted. Although the classifier correctly identified the rusty region, the background was considered more informative than the non-healthy region of the leaf. As a result, our model classifies the data by examining unrelated regions in the image. The visualization plot suggests that in its current state, our classifier is likely to associate non-healthy images with soil backgrounds. This can indicate the reasons the model has diverged. The model is suitable; the problem is the way it was fed data, which led to low test accuracy. This problem motivated us to propose a guided approach.

### 3.2. Guided Approach

The naïve approach encountered difficulty localizing only the defective region. The background soil texture was incorrectly used to distinguish between healthy and non-healthy images. Hence, a guidance system was needed to focus on the region of interest. The Guided approach was trained on segmented input images and extracted patches. Our model was guided to focus on the most important region of the image. As a result, the proposed approach achieved a validation accuracy of 98%, which is significantly higher than the 77% accuracy of the naïve approach (Table 1).

**Figure 11:**
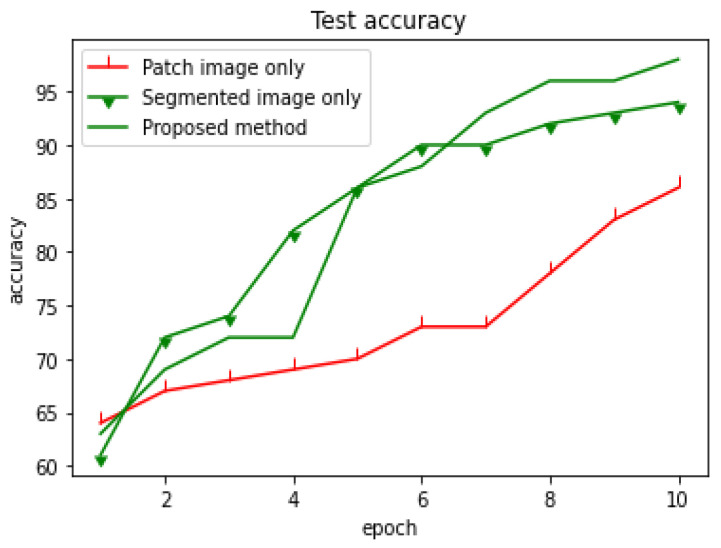
Accuracy on patch image only, segmented only, and the proposed approach (Guided approach).

We introduced two improvements with the Guided approach: using masked images without a background noise (leaves, soil, etc.) and using small patches of images with diseased regions. We carried out further experiments to determine which factor most influenced the outcome. Figure 11 demonstrates that segmenting has a greater impact, however, Training with both the segmented and segmented images achieved the highest accuracy.

**Figure 12:**
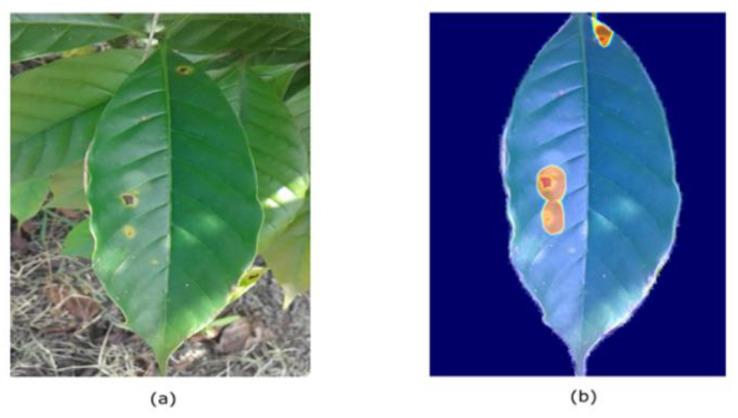
Visualization Result on Guided approach. (**a**) input image; (**b**) highlighted image.

We can remark that the guided approach is superior to the naïve approach and generalizes well on unseen data. The visualization results helped us find what went wrong in the naïve approach. Figure 12 shows the visualization result of the guided approach: Grad-CAM effectively localized the defected regions. The proposed model seems to focus mostly on the defective regions; thus, we can trust it.

### 3.3. Comparison of Visualization Methods

It is apparent from the literature review that most of the studies have targeted the problem of plant disease classification [23,24,25,26,27,28,29,30]. Nevertheless, models were considered as a black box, and it is difficult to trust a model that we can’t explain how it operates. Moreover, in disease detection and classification systems, we cannot afford to be wrong. Visualization techniques provide a way to visualize the pixels in an image that contribute the most to its classification by the model. A heatmap was generated to localize the responsible region for classification. Visualization techniques highlighted exclusively the defective regions. A visual comparison of Grad-CAM, Grad-CAM++, and Score-CAM is presented in this section. We hope this will provide insight into how to build better models for plant disease applications.

**Figure 13:**
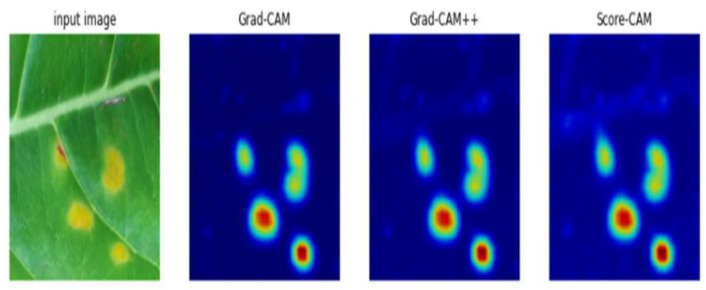
Heatmaps on patch images.

Visualization techniques were applied to the extracted patches and full-size images for comparison. Interestingly, we found that all visualization techniques effectively localized the defective region on the patches of the image. A heatmap is shown in Figure 13, the heatmap shows that Grad-CAM, Grad-CAM++, and Score-CAM effectively identify the defective regions. The dark red line on the heatmap shows the location responsible for classifying the image as an abnormal image.

**Figure 14:**
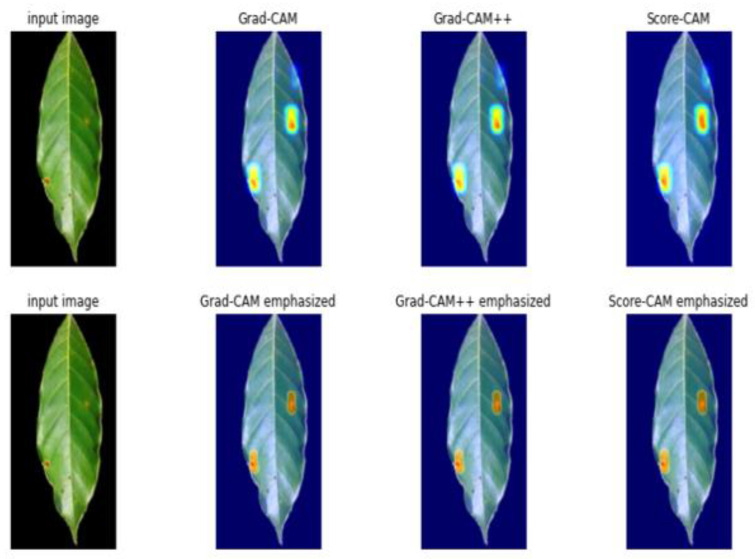
Visualization Result of Grad-CAM, Grad-CAM++, and Score-CAM.

Furthermore, we tested our model on full-size images and added heatmaps outputs to input images to generate the visualization result. In most cases, all visualization techniques successfully identified the most distinguishable region of the target object, which indicates that any of these visualizations achieve competitive results for visualization (as shown in Figure 14). The emphasized versions were identified by calculating the sigmoid of the heatmap before incorporating it into the input image. The defective area was more heavily emphasized than normal grade-CAM. Moreover, none of these methods was exposed to bounding box annotation, but they all effectively localized the defective region. This indicates that visualization can also be used for coffee disease localization in a weakly supervised manner.

**Figure 15:**
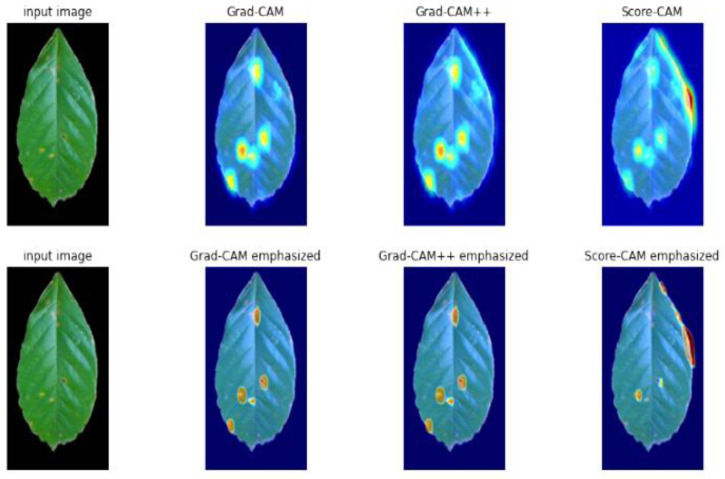
Visualization Result of Grad-CAM, Grad-CAM++, and Score-CAM.

Grad-CAM and Score-CAM both show the ability to locate defected regions, but the saliency maps of Score-CAM are more focused than those of Grad-CAM++. For this reason, occasionally, Score-CAM localizes a healthy region that resembles a diseased region. In Score-CAM, each target object with a high confidence score predicted by the model can be highlighted independently [35]. Therefore, all pieces of evidence related to the target class can obtain responses and are assembled through linear combination. In Figure 15, on the right side of the image, there is a structure that looks like a disease; however, it is not one. As the Score-CAM can focus on a small amount of detail, it highlighted the structure as a defect.

## 4. Conclusions

In this paper, we analyzed and presented the use of visualization in disease detection methods. We demonstrated the possibility of using visualization to see the effectiveness of our model in detecting diseases instead of treating disease detection systems as black boxes. Further, a classification accuracy of 98% was achieved using the guided approach. Our study utilized three visualization methods: Grad-CAM, Grad-CAM++, and Score-CAM. A visual comparison of all visualization methods for coffee disease classification was also performed. This paper is intended to provide an understanding of how to build a better model for detecting plant diseases, and we consider visualization to be a vital component.

In future work, we plan to develop a coffee disease detection and localization system. Annotating images by object bounding boxes is expensive, especially in developing countries where expertise and technology are scarce. We have already shown a practical method of localizing coffee diseases without annotating images with object bounding boxes during training. Thus, in the future, we aim to investigate a weakly supervised coffee disease detection system.

## Figures and Tables

**Table 1:** Comparisons of Naïve approach and guided approach.

Epoch	Naïve Approach	Proposed Approach (Guided Approach)
4	72%	71%
5	73%	72%
6	75%	86%
10	74%	98%
14	75%	98%

## Data Availability

The data presented in this study are openly available in Mendeley Data at doi:10.17632/c5yvn32dzg.2, reference number 36.
